# Chickpea-*Fusarium oxysporum* interaction transcriptome reveals differential modulation of plant defense strategies

**DOI:** 10.1038/s41598-017-07114-x

**Published:** 2017-08-10

**Authors:** Medha L. Upasani, Bhakti M. Limaye, Gayatri S. Gurjar, Sunitha M. Kasibhatla, Rajendra R. Joshi, Narendra Y. Kadoo, Vidya S. Gupta

**Affiliations:** 1 Biochemical Sciences Division, CSIR-National Chemical Laboratory, Dr. Homi Bhabha Road, Pashan, Pune, 411008 India; 2 Department of Microbiology, Savitribai Phule Pune University, Pune, 411007 India; 3 HPC-Medical and Bioinformatics Applications Group, Center for Development of Advanced Computing, Savitribai Phule Pune University Campus, Pune, 411007 India

## Abstract

Fusarium wilt is one of the major biotic stresses reducing chickpea productivity. The use of wilt-resistant cultivars is the most appropriate means to combat the disease and secure productivity. As a step towards understanding the molecular basis of wilt resistance in chickpea, we investigated the transcriptomes of wilt-susceptible and wilt-resistant cultivars under both *Fusarium oxysporum* f.sp. *ciceri* (Foc) challenged and unchallenged conditions. Transcriptome profiling using LongSAGE provided a valuable insight into the molecular interactions between chickpea and Foc, which revealed several known as well as novel genes with differential or unique expression patterns in chickpea contributing to lignification, hormonal homeostasis, plant defense signaling, ROS homeostasis, R-gene mediated defense, etc. Similarly, several Foc genes characteristically required for survival and growth of the pathogen were expressed only in the susceptible cultivar with null expression of most of these genes in the resistant cultivar. This study provides a rich resource for functional characterization of the genes involved in resistance mechanism and their use in breeding for sustainable wilt-resistance. Additionally, it provides pathogen targets facilitating the development of novel control strategies.

## Introduction

Chickpea (*Cicer arietinum* L.) is the third most important grain legume considering worldwide production (http://www.cgiar.org/our-strategy/crop-factsheets/chickpea/) and is a valuable source of dietary protein especially for the majority of the vegetarian population in the Indian sub-continent. However, over the past few decades, there has only been marginal increase in chickpea productivity and this is mainly attributed to various biotic (e.g. Ascochyta blight, Fusarium wilt and pod borer) and abiotic (e.g. drought, salinity, heat, etc.) stresses. Reducing the losses due to these stresses is primarily important to enhance the crop production. *Fusarium oxysporum* f.sp. *ciceri* (Foc) infects chickpea causing the vascular wilt disease, which is highly destructive and worldwide in occurrence. The disease can cause up to 90% annual yield losses^1^. It is difficult to manage the disease either through crop rotation or application of fungicides because of its soil-borne nature. Moreover, the pathogen can survive in soil for up to six years even in the absence of the host, which makes its control very difficult^2^. Hence, using wilt-resistant cultivars is the most effective and eco-friendly strategy to manage the disease.

To understand the molecular mechanisms underlying the plant-pathogen interactions, various ‘omics’ approaches are being employed. In the chickpea-Foc pathosystem, transcriptome analyses have been performed using cDNA-AFLP and cDNA-RAPD techniques, where several defense related genes of chickpea as well as virulence related genes of Foc were detected^3^. Further, high throughput sequencing has identified wilt responsive microRNAs involved in regulating plant development and pathogen growth by acting as positive or negative regulators, depending on their target genes^4^. Specifically, these studies revealed activation of primary metabolism during the interplay between the fungus and the host^5^. Recently, we employed microscopic, proteomics and metabolomics approaches to characterize the chickpea-Foc interaction at molecular level. The microscopic approach revealed differential colonization of Foc in resistant and susceptible chickpea cultivars, wherein the resistant host severely repressed pathogen growth; while the pathogen could successfully proliferate and reproduce only in the susceptible cultivar. The proteomics and metabolomics approaches highlighted up-regulation of several metabolic pathways such as biosynthesis of flavonoids, isoflavonoids, phenylpropanoids, etc. in the resistant cultivar^6–8^.

Characteristic gene expression essential for lifestyle transitions in various phytopathogens has been deciphered using comparative genomics and transcriptomics studies^9–12^. Particularly in the genus *Fusarium*, the ‘core’ genome has been shown to be responsible for primary metabolism and reproduction, while the ‘adaptive’ genome codes for pathogen virulence, host specialization and other functions. *Fusarium oxysporum* (Fo) pathogenic to tomato and pea have the virulence and host specialization genes located on the ‘pathogenicity’ chromosomes possessing the ability to transform a non-pathogenic strain into a pathogenic one^13^. Genome analysis of Fo infecting banana revealed a large set of putative virulence associated genes required for diverse biological processes aiding pathogenesis. Similarly, transcriptome analysis highlighted significant differences in transcriptional responses between vegetative growth stage and *in planta* propagation of the pathogen^12^.

In the present study, significant transcriptional changes in both susceptible and resistant chickpea cultivars upon Foc inoculation were revealed using the LongSAGE approach^14^ coupled with next generation sequencing. Additionally, several pathogen genes with peculiar expression in both the chickpea cultivars were also identified. The expression patterns of some of these genes during disease progression were validated using quantitative reverse transcription polymerase chain reaction (qRT-PCR). We found that in the resistant cultivar, certain biological processes were characteristically activated, which provided definite advantage to it against the pathogen. While in the susceptible cultivar, the pathogen modulated the expression of a majority of the plant genes to support its own establishment, growth and proliferation.

## Results

### Analysis of LongSAGE libraries

Plants of two chickpea cultivars JG62 (wilt-susceptible; referred as ‘JG’) and Digvijay (wilt-resistant; referred as ‘DV’) were either inoculated (‘I’) with the pathogen (JGI and DVI), or mock-inoculated (‘C’) with sterile de-ionized water (JGC and DVC) as described in the Methods section. The results of tag mapping of the four libraries (JGI, DVI, JGC and DVC) are presented in Table 1. Among the four LongSAGE libraries, the highest number of tags (386458) was obtained in DVI library followed by JGC (189947). However, the number of mapped tags was highest in JGC followed by DVI library. Similarly, DVI had the highest number of mapped transcripts, followed by JGC. Interestingly, the JGI library showed the least number of tags, mapped tags and mapped transcripts.

**Table 1:** Statistics of tag mapping of the four LongSAGE libraries.

Library	Total no. of tags	No. of mapped tags	Total no. of mapped transcripts	Total no. of transcripts with unique matches*	Total no. of transcripts with selected matches^^^
JGC	189947	37253	14658	5872	8786
DVC	189703	33497	14534	6270	8264
JGI chickpea	149785	27128	12581	6085	6496
JGI Fusarium		1697	1368	1095	273
DVI chickpea	386458	34380	14783	5861	8922
DVI Fusarium		278	265	251	14

^*^‘Unique’ match represents those tags mapped only on single transcript.

^^^‘Selected’ match represents multiple tag sequences mapped on the same transcript.

### Characteristic Differentially Expressed Genes and their functional classification

Differential gene expression (DGE) sets defined by comparisons across all the four LongSAGE libraries revealed differentially and uniquely expressed genes (p-value < =0.05 and FDR (False Discovery Rate) < =0.05) within the compared pair of libraries (Tables 2 and S1, S2, S3 and S4). Transcriptome analysis revealed the highest number of 3816 DEGs in DE_JGC_JGI excluding fungal sequences (unique to JGI library); wherein 43.16% genes were down-regulated (1647) and 32.23% (1230) were up-regulated. Alternatively, in DE_JGC_DVC, 2987 DEGs were obtained, with down-regulated genes (36.55%) accounting slightly higher than the up-regulated (31.36%) ones.

**Table 2:** Differential gene expression analysis.

DGE sets	No. of differentially expressed genes	Up-regulated genes	Down-regulated genes	Significantly expressed genes in only one Library
DE_JGC_JGI (CA genes)	3816	1230	1647	695 (JGC), 256 (JGI)
DE_DVC_DVI (CA genes)	3429	1390	1100	312 (DVC), 349 (DVI)
DE_JGC_DVC (CA genes)	2987	937	1092	77 (JGC), 68 (DVC)
DE_JGI_DVI (CA genes)	3622	1694	1106	480 (JGI), 904 (DVI)
DE_JGI_DVI (FO genes)	NIL	NIL	NIL	533 (JGI), 5 (DVI)

Differential Gene Expression (DGE) Sets represent comparisons among the four SAGE libraries. For example, DE_JGC_JGI shows DEGs up- or down-regulated in JGI compared to JGC. CA stands for *Cicer arietinum* (chickpea) and FO stands for *Fusarium oxysporum*.

The annotation tool ‘Mercator’ (http://www.plabipd.de/portal/mercator-sequence-annotation) allowed assignment of genes of all the four sets into 35 functional classes referred to as ‘bins’^15^. They were analyzed based on the most frequent bins assigned and their regulation. Fig. S1 shows the distribution of 24 major bins among the up- and down-regulated genes in the four comparisons. Based on these assignments, an attempt was made to understand the role of the DEGs in modulating the cellular mechanisms in chickpea in response to Foc attack. The MapMan annotation tool was used to display DEGs from three sets *viz*. DE_JGC_JGI, DE_DVC_DVI and DE_JGI_DVI with respect to stress^15^ (Fig. S2). Overall, higher number of stress responsive transcripts was observed in JGI. However, up-regulated genes in response to fungal attack were more in DVI. The transcripts associated with abiotic stress, ROS homeostasis, heat-shock proteins, secondary metabolism and PR proteins were noticeably up-regulated in DVI as compared to those in DVC and JGI. Interestingly, reticulon-like protein B2 (RTNLB2), showed highest expression (15.36 fold) in DVI. Transcripts belonging to ROS homeostasis were peroxidase, glutathione-S transferase, catalase, glutaredoxin and thioredoxin family proteins. The PR protein category particularly included disease resistance proteins (DRR) SR1, DRR 206 and TMV resistance protein. Likewise, several heat shock proteins (HSPs) were highly expressed in DVI. Moreover, lignin biosynthetic enzymes such as 4-coumarate–CoA ligase (4CL), cinnamoyl-CoA reductase 1 (CCR1) and caffeoyl-CoA O-methyltransferase (CCoAOMT) were also up-regulated in DVI. MapMan revealed a disease resistance protein belonging to nucleotide-binding site leucine-rich repeat (NBS-LRR) gene family in DE_DVC_DVI set.

### Clustering of core DEGs across the comparisons

**Figure 1:**
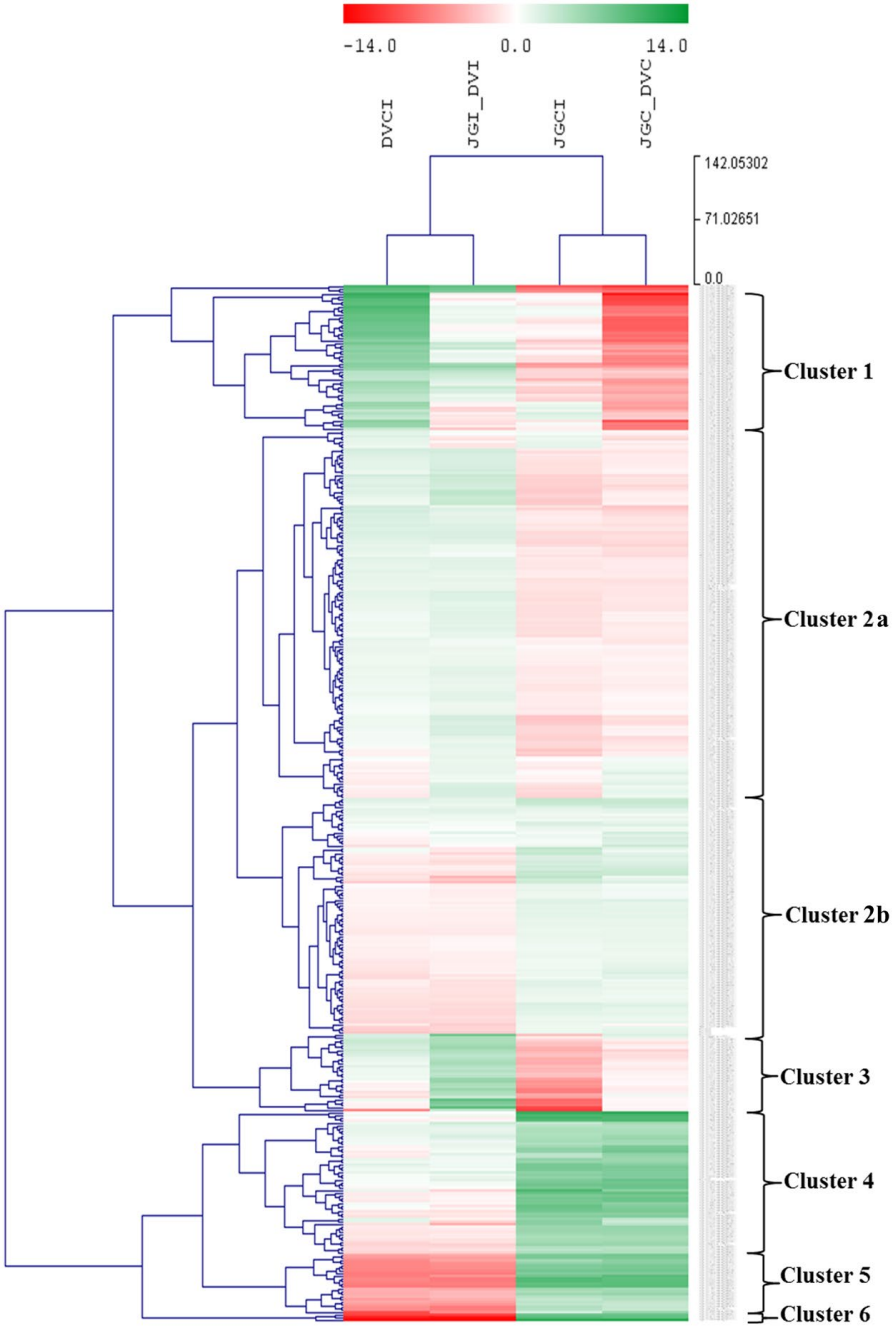
Heatmap and cluster analysis of core DEGs (chickpea) across four datasets. Comparison of significant DEGs among DGE sets resulted in 400 core DEGs (all having LFC ≥1 in at least one of the sets). Heatmap was generated with the Log2fold change (LFC) values. Column one represents DE_DVC_DVI (DVCI), column two represents DE_JGI_DVI (JGI_DVI), column three represents DE_JGC_JGI (JGCI) and column four represents DE_JGC_DVC (JGC_DVC). Each row represents corresponding genes with their identities. The list of genes in each cluster is provided in Table S5. Up-regulation and down-regulation is indicated by color change from pale to dark red (−14 LFC) and green (+14 LFC) with white (0 LFC) representing no change in expression. These genes were clustered using Euclidean distance and complete linkage method.

To identify the genes responsive to pathogen inoculation either in the resistant or susceptible cultivars, we compared significant DEGs above or below the basal gene expression level between the two cultivars among all the four DGE sets, where the genes with LFC (Log2fold Change) <1 in all the four sets were omitted. A total of 400 DEGs (all having LFC ≥1 in at least one of the sets) were clustered into six expression patterns (clusters 1–6) based on hierarchical clustering (Fig. 1 and Table S5). Cluster 1 included the genes mainly belonging to protein metabolism, RNA-regulation of transcription, hormone metabolism (Gibberellin and Jasmonate), calcium signaling and stress (biotic and abiotic). These genes were highly up-regulated in DVI; for example, ubiquitin-conjugating enzyme E2 28 (~20 fold up-regulation), peroxidase 42 (~18 fold up-regulation), glutathione S-transferase (~18 fold up-regulation) and ADP ribosylation factor (ARF) (~15 fold up-regulation). Cluster 2a represented the genes belonging to protein synthesis and degradation, RNA-regulation of transcription, signaling (calcium, G-proteins, MAP kinases and LRR) and stress. This group was down-regulated in JGI and up-regulated in DVI, and included 14-3-3-like protein B, heat shock proteins, calcineurin and serine-threonine protein kinase etc.

Alternatively, the genes in cluster 2b showed the opposite trend i.e. up-regulation in JGI and down-regulation in DVI. This included transcripts pertaining to protein synthesis and degradation, secondary metabolism and hormone metabolism such as ethylene ACC oxidase, ethylene responsive transcription factor RAP 2–1, isoflavone-7-O-methyltransferase 9, flavonoid 3′-monooxygenase, isoflavone 2′-hydroxylase, NAD(P)H-dependent 6′-deoxychalcone synthase, expansin like genes etc. The cluster 3 exhibited an interesting expression profile, depicting candidates with reduced basal gene expression in DVC as compared to JGC. Few of these genes were highly down-regulated in JGI as compared to JGC and up-regulated in DVI with respect to DVC. This finally reflected as much higher up-regulation of these transcripts in DVI as compared to JGI. The genes belonging to this cluster represented protein metabolism, RNA-regulation of transcription, G-protein signaling, aromatic amino acid synthesis and stress mechanisms such as auxin-binding protein ABP19a, phospho-2-dehydro-3-deoxyheptonate aldolase, chitinase and glucanase, etc.

Cluster 4 genes were up-regulated in DVC and JGI than those in JGC. These involved processes like protein metabolism, brassinosteroid hormone metabolism, C3H zinc finger regulation of transcription and stress, and genes like aquaporin PIP-type 7a, magnesium protoporphyrin IX monomethyl ester [oxidative] cyclase (MPP) etc. The cluster 5 had much higher up-regulation in DVC and JGI than JGC, while down-regulation in DVI as compared to JGI and DVC. These mainly included the genes belonging to photosynthesis, protein synthesis and hormone metabolism such as transcripts like sedoheptulose-1,7-bisphosphatas, ferredoxin, linoleate 9S-lipoxygenase etc. Finally, the cluster 6 included the genes exhibiting similar pattern as that of cluster 5 and belonging to N-metabolism and photosynthesis such as chlorophyll a-b binding protein, ferredoxin-nitrite reductase etc. The evaluation of DEGs by BLASTP analysis against the PRGdb (plant resistance genes database)^16^ revealed the presence of 15 resistance genes from the set of 400 DEGs, a majority (10) of which belonged to cluster 2; while three belonged to cluster 4 and two belonged to cluster 5. More resistance genes were expressed in DVI than in JGI (Table S6).

### Interaction network of DEGs

**Figure 2:**
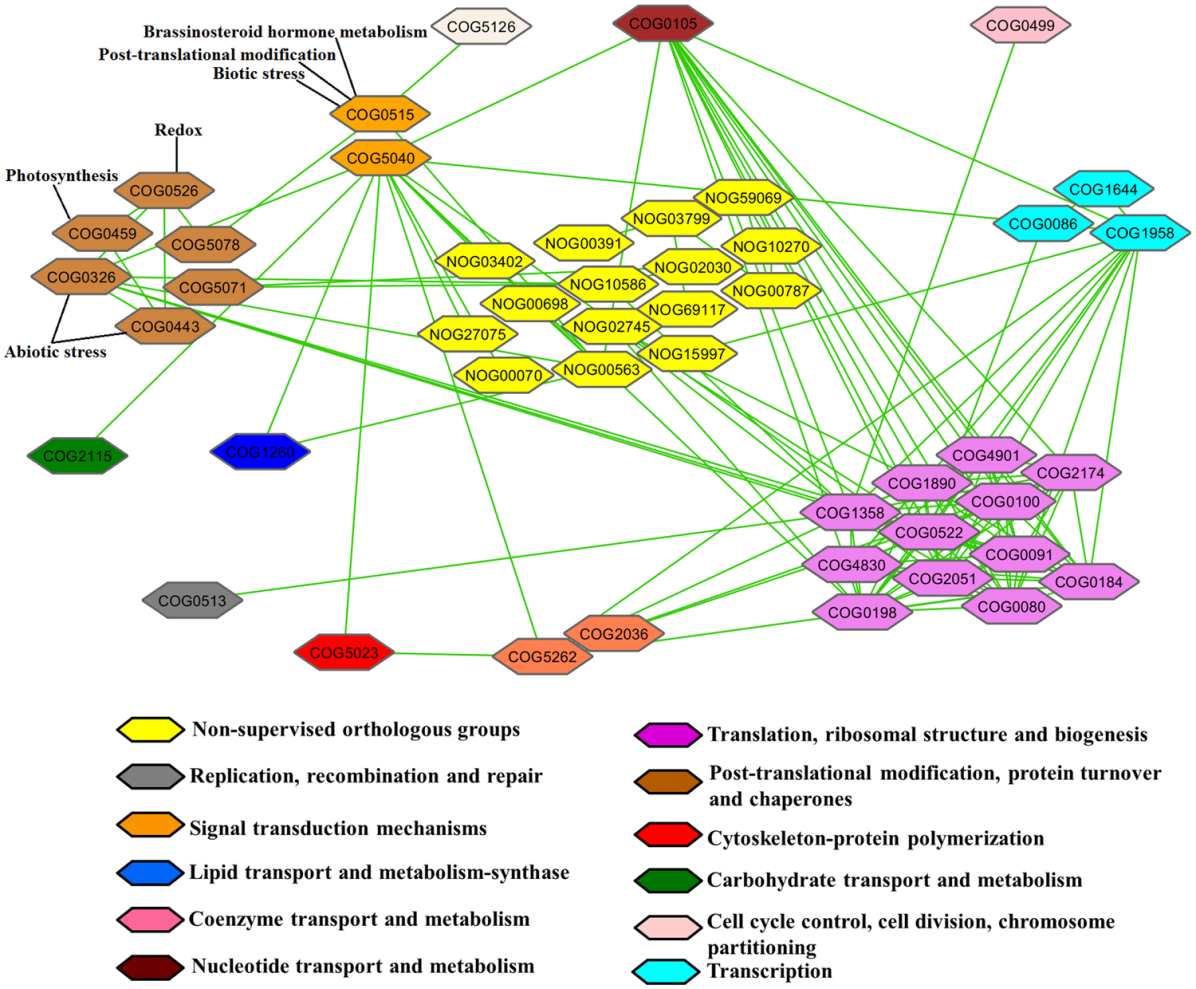
Protein-protein interaction network analysis (PPI) of core DEGs. PPI analysis was conducted using STRING (version 10.0, http://string-db.org, COG mode) and *Glycine max* as an organism (nearest neighbor legume in the organism list present in STRING database). The confidence score was set at ≥0.70 and co-expression and experiment parameters were chosen. COG descriptions along with color codes are mentioned in the figure.

To determine the interactions of these DEGs, protein–protein interaction (PPI) analysis was performed using STRING (http://string-db.org). Fifty-seven best assigned COGs (Clusters of Orthologous Groups), representing 62 unique DEGs, obtained based on most significant E-value using *Glycine max* as the organism (nearest neighbor legume in the STRING database), were used to construct an interaction network (Fig. 2). The PPI network of all the DEGs was extracted from the whole interaction network and reconstructed using Cytoscape. The PPI network highlighted several protein functional groups interacting with each other. Majority of the COGs (21.05%) belonged to ‘Translation, ribosome structure and biogenesis’, which showed maximum interactions with other groups followed by ‘Post-translational modification, protein turnover and chaperones’. Mercator terms assigned to the DEGs and the 57 COGs associated with these DEGs shared the same biological functions (Table S7). However, as depicted in Fig. 2, a group of COGs represented additional biological functions apart from the COG descriptions. For example, the ‘Signal transduction mechanisms’ COG contained an additional set of DEGs assigned to Mercator terms like ‘brassinosteroid hormone metabolism’, ‘post-translational modification’ and ‘biotic stress’. Similarly, ‘Post-translational modifications’ COG possessed DEGs with additional Mercator terms like ‘photosynthesis’, ‘redox’ and ‘abiotic stress’.

### Genes exclusively expressed in JGI and DVI

The genes expressed uniquely in either of the cultivars upon inoculation were analyzed. In JGI, these genes (562; Table S8) might represent the candidates reprogrammed by the pathogen for its own benefit. Whereas in DVI, these genes (860; Table S9) might activate the defense response against the pathogen. The uniquely expressed important genes in DVI included beta-D-xylosidase 7, rhamnogalacturonate lyase B, pectate lyase 12, thiamine pyrophosphokinase, dirigent proteins, etc.; while the unique ontologies included cell wall and LRR (Leucine rich repeat) proteins, cofactor and vitamin metabolism, thermospermine (TSpm) synthesis, abscisic acid and cytokinin metabolism. Similarly, the uniquely expressed important genes in JGI were MLO like transcript, actin depolymerizing factor (ADF) 5 and tonoplast intrinsic protein (TIP) aquaporin type alpha, etc.; while other ontologies were shared with DVI. The uniquely expressed genes were also evaluated for the presence of R genes using the PRGdb database, which identified 52 and 45 R genes in DVI and JGI, respectively. Assessment of distribution of R protein types indicated higher proportion of NBS-LRR types (19.23%) in DVI and RLK (receptor like kinase) type R proteins (31.11%) in JGI dataset (Tables S6, S8 and S9).

### General features of the Foc transcriptome

Comparative transcriptome analysis of JGI and DVI libraries revealed a total of 1569 genes exhibiting high homology to Fo and *Fusarium graminearum* (Fg). As expected, these genes were not detected in control libraries. A total of 533 Foc transcripts were expressed only in JGI, of which 382 (71.66%) were annotated using the available resources (Table S10). These 382 transcripts were categorized into 41 functional groups belonging to three gene ontology (GO) categories: Cellular Components (CC), Molecular Functions (MF) and Biological Processes (BP) (Fig. S3). In the CC category, maximum transcripts were from ribosome and protein complex, while few were also localized to nucleus, integral component of membrane and mitochondrial part. In the MF category, the highest number of transcripts was from structural component of ribosome followed by those showing ATP binding and metal ion binding activities. Few transcripts were also involved in transferase activity, protein binding and nucleoside-triphosphatase activity. DNA binding, GTP binding, cofactor binding, oxido-reductase activity and translation initiation factor activity were presented by some of the transcripts.

In the BP category, the largest number of transcripts was involved in oxidation-reduction process and organonitrogen compound metabolic process. The remaining transcripts were involved in different cellular, metabolic processes, response to stimulus, stress, intracellular transport as well as biological regulation. KEGG pathway enrichment analysis of these genes was also performed. A total of 43 transcripts were allocated to 24 KEGG pathways (Table S11). The pathways involving the highest number of transcripts were TCA cycle (4, 10.81%), carbon fixation in photosynthetic organisms (4, 10.81%), fructose and mannose metabolism (3, 8.1%), pyruvate metabolism (3, 8.1%) and carbon fixation pathways in prokaryotes (3, 8.1%). Similarly, only five Foc transcripts were expressed exclusively in DVI, *viz*. Med A homologue, TKL protein kinase, putative tartrate transporter, etc. Eighteen Foc transcripts expressed significantly in both JGI and DVI (Table S10). Among these were the transcripts with similarity to serine rich protein, glucosidase, heat shock proteins, histone proteins and five transcripts with possible involvement in fungal growth such as tropomyocin 1, polarized growth protein rax2, woronin body major protein, fimbrin and phosphatidate cytidylyltransferase.

The fungal transcripts identified in this study were searched against the PHI database (Pathogen–Host Interactions database, http://www.phi-base.org/), which is a collection of fungal pathogenicity genes validated using gene knockout experiments. Homologues of Foc transcripts having an effect on pathogenicity in other fungal systems were identified. A Foc transcript (identified as TKL protein kinase) expressing only in DVI, was recognized as a virulence factor. Three of the eighteen Foc transcripts (ATP synthase subunit alpha and two hypothetical proteins) and 85 Foc transcripts that expressed only in JGI, showed homology to experimentally proven virulence factors. In addition InterProScan analysis was performed to gain insight into specific functions of the genes and to support functional annotation (Table S10).

### Validation of expression patterns by qRT-PCR analysis

To validate the results of LongSAGE transcriptomics and comparative analysis, eight plant genes and eight pathogen genes were used for verifying their expression pattern at time-points 0, 16, 24 hpi and 2, 4, 7, 14 and 28 dpi in root tissue and 24 hpi, 7 and 14 dpi in shoot/stem tissue. The relative expression levels indicated by LongSAGE results were reflected in qRT-PCR. For example, the chickpea genes 14-3-3, auxin binding proteins ABP19a and mitogen activated protein kinase, which showed higher expression in DVI in LongSAGE analysis, also showed higher fold changes at several time-points in DVI in qRT-PCR analysis. Similarly, several Foc genes also showed higher fold change in JGI by qRT-PCR analysis (Figs S4, S5, S6 and S7).

## Discussion

Gene expression profiling upon biotic stress has been broadly studied in several plant species using a variety of transcriptomics approaches^17^. In the present study, comparison of four LongSAGE libraries elucidated key factors involved in chickpea resistance mechanisms upon Foc inoculation. The four DGE sets revealed several biological processes induced in resistant cultivar upon inoculation, which otherwise were inactive in absence of the pathogen. Alternatively, several biological processes were repressed in susceptible cultivar upon fungal inoculation, indicating that the pathogen might govern the host metabolic machinery for its own benefit. Among these, important biological processes that were highlighted in the functional categories of the DGE sets are discussed below.

An interesting feature in the transcriptome analyses was ‘protein synthesis, degradation and post-translational modifications’ represented the most abundant functional class among all. PPI network analysis also showed higher abundance of these proteins with both intra- and inter-connections with other Foc induced proteins. In the resistant cultivar, significant up-regulation of ribosomal proteins (60S, 40S and 50S), ubiquitin-conjugating enzyme E2 and protein kinases suggested that protein synthesis plays an important role in disease resistance; wherein ubiquitination has already been suggested to be a crucial contributor of plant innate immune response^18, 19^. Increasing evidences have shown that many key components of plant disease resistance undergo protein degradation in response to pathogen infection for mounting defense hypersensitive response (HR) and Systemic Acquired Resistance (SAR)^20–22^.

‘Signaling’ was another notable functional class with high transcript abundance in DVI. It mainly included calcium, G-proteins and light induced signaling, followed by Receptor Like Kinases (RLKs) and Mitogen Activated Protein (MAP) kinases based signaling. As an important secondary messenger in plant cells, changes in Ca^2+^ concentration were detected during effector-triggered immunity (ETI), specifically in the incompatible interactions between *Pseudomonas syringae* pv. *tomato* containing *avrRpm1* and *RPM1* in *Arabidopsis* ^23, 24^. Heterotrimeric G-proteins, well known in stress signaling^25^, have been proposed as activators of plant cell death and mediators of stomatal closure signaling^26^ as well as are involved in cell wall biogenesis/metabolism and ABA signaling^27, 28^. The induction of G-protein signaling in DVI suggested its function in early defense response against Foc. In plants, RLKs have diverse functions, such as development, growth, hormone perception and the response to pathogens. In addition to general elicitor recognition, RLKs with LRR motifs participate in the recognition of pathogen avirulence factors (Avr genes) produced by specific strains of plant pathogens^29^. We found that 27 RLK transcripts were up-regulated in DVI. RLK regulation has also been linked to ubiquitination as a means of targeting receptors for degradation to mitigate plant immune response^30^. Thus, the role of receptor kinases and Ca^2+^ mediated signaling in conjunction with ubiquitin-conjugating enzyme E2 was suggestive of providing the resistant cultivar a definite advantage in mounting defense response against the pathogen. The role of MAP kinases in plant defense upon pathogen associated molecular pattern (PAMP) treatment^31^ and in response to insect pests^32^ has also been reported. Up-regulation of MAP kinases 3, 5, 16 and RALF (Rapid alkalization factor) like 33 in resistant inoculated plant in our study, affirmed their role in plant defense.

Another important functional class up-regulated in DVI was related to hormone metabolism. The plant hormones ethylene, jasmonic acid (JA), and salicylic acid (SA) play crucial roles in plant growth, defense and response to environmental cues. Similarly, the role of other plant hormones, such as auxins, abscisic acid (ABA), cytokinins, gibberellins and brassinosteroids, in plant immunity has also been reported^33^. Up-regulation of the transcripts related to several phytohormones particularly auxin biosynthesis (especially *GH3*.*6* like) in the present study, positively correlated with the published reports of increased resistance to pathogen^34, 35^. Similarly, up-regulation of the genes related to cytokinin homeostasis indicated their key roles in structuring the plant defense response. Previous reports in *Arabidopsis* have shown the importance of cytokinin homeostasis (cytokinin synthases, dehydrogenases and glycosyltransferases) in imparting resistance to *Verticillium longisporum* ^36, 37^. ABA is another phytohormone, which is a complex modulator of plant defense responses as shown in *Arabidopsis* ^38, 39^. We found that several transcripts associated with ABA synthesis, degradation and signal transduction were also up-regulated in DVI, which indicated their key roles in chickpea defense. Transcripts for several pathogenesis related proteins, Major latex proteins^40^, auxin binding proteins^41^, etc. from ‘biotic and abiotic stress response’ category were detected with higher abundance and elevated expression in DVI. These proteins provided R gene mediated response, strong lignification, proteinase inhibitory activities and chaperon like functions enriching the defense response of resistant cultivar against Foc.

**Figure 3:**
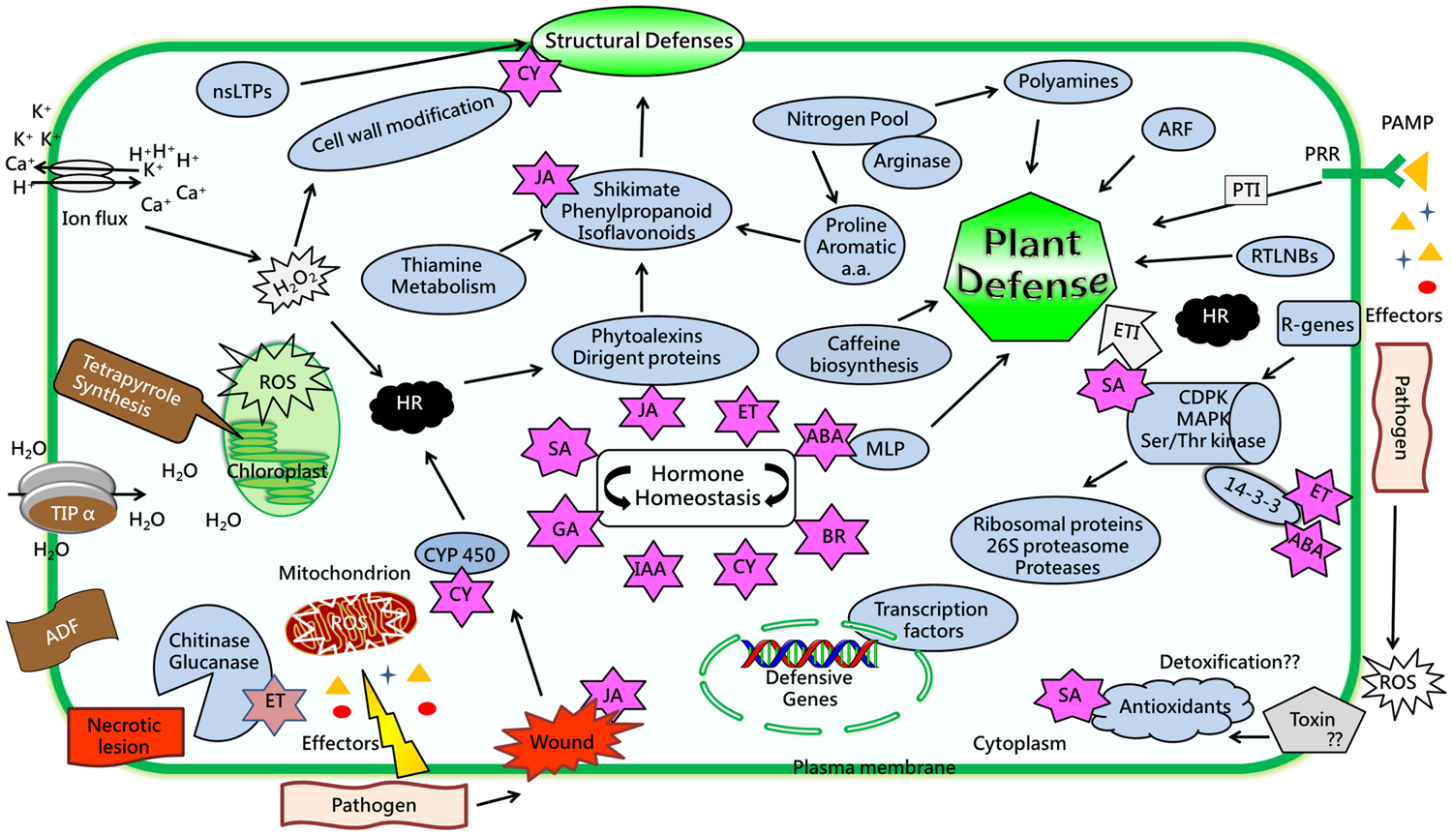
Schematic representation showing the interconnections of different biological processes induced in chickpea. Representation shows cell processes contributing to defense response in resistant cultivar (Blue and green color) with few processes that might render weakened response in susceptible cultivar (brown color).

A schematic representation of transcriptome comparisons and PPI network of cell responses contributing to plant defense in resistant cultivar in correlation to the available literature is depicted in Fig. 3. It appears that Foc inoculation in resistant chickpea cultivar triggers ROS production leading to SA production, which plays a crucial role in maintaining redox homeostasis through antioxidant activity. Several factors like non-specific lipid transfer proteins (nsLTPs), CYP450, dirigent proteins and phytoalexins are important in generating defensive shield over plant surfaces and thus contribute to successful structural defense. In addition, the expression of signaling components and pathogenesis related proteins in resistant cultivar gives the plant an advantage in mounting prompt defense. Conversely, up-regulation of certain factors such as actin depolymerization factor (ADF), aquaporins, tetrapyrrole synthesis, etc. in JGI adds to its susceptibility. Thus, the key difference between the resistant and the susceptible cultivars was timely detection of the invading pathogen and rapid and immediate activation of defense responses in response to pathogen effectors in the resistant cultivar.

**Figure 4:**
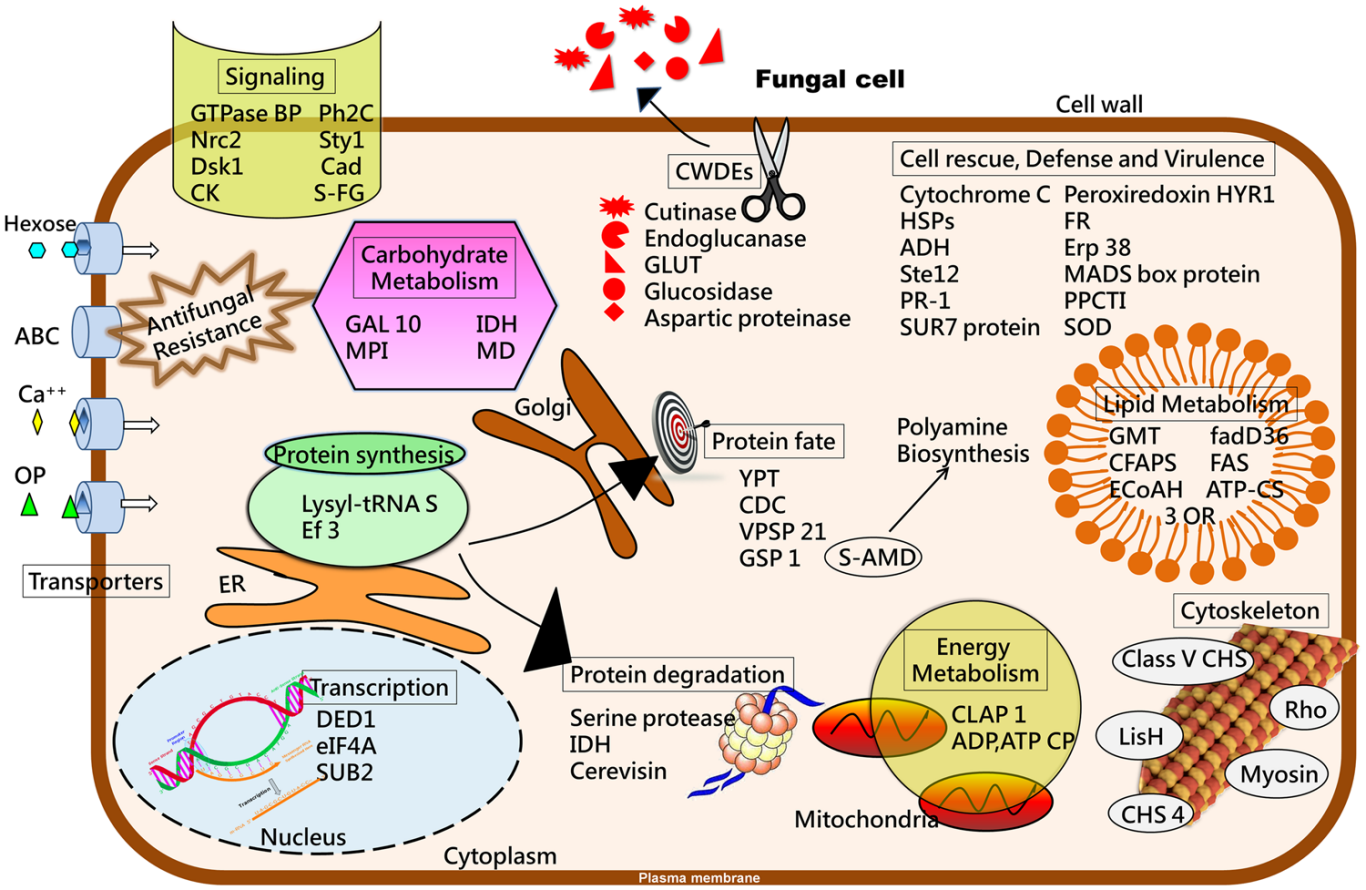
Schematic overview of Foc metabolism during pathogenesis in the susceptible host. The overview shows several aspects of Foc metabolism operational in the susceptible host based on the transcriptomics and its functional classification. The presentation mainly includes the genes, expressed only in JGI, homologues of which are proven virulence factors in PHI database (Pathogen-Host Interaction).

Alternatively, comparative analysis of transcriptomes of both the cultivars challenged by Foc revealed that a large number of Foc genes expressed only in JGI (533), while only five Foc genes could express in DVI, substantiating the strong defense strategy of DV. Based on the transcriptome analysis and further functional categorization, a schematic overview of Foc metabolism that might be operational during pathogenesis and successful disease establishment in JG62 has been presented in Fig. 4. It includes the genes expressed only in JGI that are homologues of known virulence factors based on PHI database. As depicted in the figure, almost all the biological processes required for fungal invasion, growth and pathogenesis were active in JGI. Among these processes, plant cell wall degradation mediated by cutinase, endoglucanase, 1,3-beta-glucanosyltransferase, glucosidase and aspartic proteinase, has shown to be an important virulence mechanism. We also identified several Foc transcripts related to signal transduction. Since signal transduction cascades mediate communication between environmental signals and the cellular machinery controlling growth and differentiation, expression of various kinases like serine/threonine protein kinases and protein phosphatases only in JGI might have accelerated fungal colonization. Further, several Foc transcripts involved in cell rescue, defense and virulence were expressed only in JGI. This reveals that in the susceptible cultivar, the fungus could successfully evade the plant defense responses and proliferate. In addition to these, all the basic metabolic processes of the fungus including carbohydrate, protein, lipid, energy and cytoskeleton related metabolism, were functional only in JGI. This indicated that the pathogen successfully hijacked the host metabolic machinery for its proliferation and reproduction. Alternatively, the resistant cultivar Digvijay successfully suppressed the expression of most of the fungal genes, quickly arresting the pathogen after invasion and preventing its further proliferation and reproduction.

In summary, this study provides valuable insights into the molecular interactions between chickpea and *Fusarium oxysporum* f.sp. *ciceri* by identifying several known as well as novel genes. The study revealed that in the resistant cultivar, the transcripts related to lignification, hormonal homeostasis, plant defense signaling, R-gene mediated defense, etc. contributed to successful defense against Foc. While in the susceptible cultivar, the transcripts related to photosynthesis, actin depolymerization, etc. supported pathogen proliferation. Interestingly, several Foc genes were expressed only in the susceptible cultivar and suppressed severely in the resistant cultivar. The results of the present study are in accordance with our earlier report, wherein confocal imaging and quantitative polymerase chain reaction (qPCR) revealed substantially less pathogen load in the resistant cultivar. Functional characterization of these chickpea and Foc transcripts would yield important clues to the resistance mechanism and also provide vital Foc targets for crop improvements through genetic engineering.

## Methods

### Plant inoculation assays

Two chickpea cultivars JG62 (wilt-susceptible; referred as JG) and Digvijay (wilt-resistant; referred as DV) were used for the study. Seeds of the cultivars were surface-sterilized using 1% sodium hypochlorite and soaked overnight in sterile deionized water. They were wrapped in wet sterile muslin cloth till sprouting and transferred to surface-sterilized plastic cups containing autoclaved Soil Rite (mixture of 75% Irish Peatmoss and 25% horticulture grade Expanded Perlite; obtained from M/s Naik Krushi Udyog, Pune, India). The plants were grown for one week in growth chamber (14 h light/10 h dark, 22–25 °C, 50–60% relative humidity) and inoculated with standard Foc races 1, 2 and 4 as described in our previous study^8^. Seedlings treated with sterile deionized water served as controls. Pathogen inoculated treatments were designated as JGI and DVI, while the controls were designated as JGC and DVC. The root and shoot/stem tissues from both the cultivars were collected separately for each Foc race at 11 time points *viz*. 0 hours post inoculation (hpi), 8 hpi, 16 hpi, 24 hpi, 2 days post inoculation (dpi), 3 dpi, 4 dpi, 7 dpi, 14 dpi, 21 dpi and 28 dpi. The pathogenicity assays were conducted in triplicates as depicted in Fig. S8.

### Construction of LongSAGE libraries

Total RNA was isolated from 100 mg chickpea root and shoot/stem tissues from both resistant and susceptible cultivars, separately for each time-point using the Spectrum Plant Total RNA isolation kit (Sigma-Aldrich, USA). It was reverse transcribed using the High-Capacity cDNA Reverse Transcription Kit (Applied Biosystems, USA). Total RNAs from all the 11 time-points of individual Foc races (1, 2 and 4) were normalized using the chickpea reference gene *Actin*. Based on this normalization, RNAs from all these time-points were pooled for control and pathogen challenged plants separately and four LongSAGE libraries (JGC, JGI, DVC and DVI) were constructed using the I-SAGE^TM^ Long Kit (Invitrogen, USA) as depicted in Fig. S9.

### Analysis of LongSAGE libraries

The four LongSAGE libraries were sequenced using the 318 chip and the Ion-Torrent PGM system (Genotypic Technologies, Bangalore, India). The raw data in the fastq format were processed with the FastQC toolkit (http://www.bioinformatics.babraham.ac.uk/projects/fastqc/) to remove shorter and low-quality reads. The high quality reads were used for extracting the ditags. A ditag is defined as the 32–38 bp stretch of nucleotides flanked between two ‘CATG’ sequences. The ditags thus obtained were split into individual tags of 16–19 bp using in-house developed Perl scripts. Reverse complements of tags ending with ‘CATG’ were also considered in the final dataset of tags. Tag mapping analysis was performed using the SeqMap tool with one mismatch allowed^42^ for ‘unique match’ (a tag which mapped uniquely to only one transcript) and ‘selected match’ (more than one tags matching on a single transcript, wherein the tags mapping towards the 3′ end of the transcripts were considered). The frequency of ‘unique match’ tag was assigned to the transcript as a measure of its expression value, while the frequencies of all ‘selected match’ tags mapping on single position were summed up and the frequency values thus obtained were assigned to the transcript. Only the tags having frequency of more than 1 were considered for further analysis. This entire tag extraction and mapping procedure was performed separately for both plant and fungal tags using the respective reference transcriptomes. The databases used for mapping are indicated in Table 3.

**Table 3:** Databases used for mapping SAGE tags in transcriptome analysis.

SAGE tags from	Reference transcriptomes used for mapping (Designation)	Source
Chickpea	*Cicer arietinum* L (CA)	NCBI (Release 197; Aug 2013)
	*Medicago truncatula* (MT)	Ensembl Plants (Database version 87.2; September 2013)
	*Glycine max* (GM)	NCBI (Release 197; Aug 2013)
	*Cajanus cajan* (CC)	NCBI (Release 197; Aug 2013)
	*Lotus japonicus* (LJ)	NCBI (Release 197; Aug 2013)
*Fusarium oxysporum*	*Fusarium oxysporum* (FO)	Ensembl Fungi (Database version is 87.2; Sept 2013)
	*Fusarium graminearum* (FG)	Broad Institute (March 2007)

### Differential gene expression analysis

After mapping, raw tag counts for the individual libraries were obtained. These raw counts were normalized and analyzed for differential gene expression resulting into four datasets *viz*. DE_JGC_JGI, DE_DVC_DVI, DE_JGC_DVC and DE_JGI_DVI. For example, DE_JGC_JGI represents the differentially expressed genes (DEGs) between JGC and JGI libraries. The TMM (trimmed mean of M-values) method^43^ was used for normalization of the datasets using the edgeR package. Gene expression analyses were performed using the combined approach of the Audic and Claverie test (ACT) and Chi-square test (Chi)^44, 45^. The p-value of significance obtained was adjusted to reduce the false discovery rate (FDR)^46^. The cutoffs used for significantly expressed genes were p-value ≤ 0.05 and FDR ≤ 0.05. The normalized values were used to find the Log2fold change (LFC) for each transcript in all the four datasets. The DEGs were obtained by comparing all the four libraries (Table 2). The up-regulated and down-regulated genes were selected using cut-off of 2 fold (LFC ≥ 1). The DEGs from plant species were processed for gene enrichment analysis using the Mercator tool^47^. Further, MapMan analysis^15, 48^ was performed for pathway enrichment of the DEGs. Cluster analysis of the DEGs was performed by employing the Euclidean distance method over a complete linkage using MeV (MultiExperiment Viewer)^49^. The DEGs from the pathogen were processed for gene enrichment analysis using Blast2GO^50^.

### Bioinformatics analysis of chickpea and Foc DEGs

A total of 400 chickpea DEGs were obtained across the four datasets and used for BLASTX analysis using the NCBI nr database to retrieve corresponding protein sequences based on E-value and bit score. The best assigned Clusters of Orthologous Groups (COGs) for these proteins were selected from *Glycine max* and used for protein-protein interactions (PPI) analysis using STRING (Search Tool for Retrieval of Interacting Genes/Proteins) database (version 10.0, http://string-db.org) in COG mode. The assigned COG descriptions were obtained from EggNOG 4.5 (http://eggnogdb.embl.de/#/app/home). Only the interactions with confidence score of ≥0.7 and based on co-expression and experiment conditions were used to construct the network, which was displayed using Cytoscape (version 3.3.0) (http://www.cytoscape.org/). The annotated Foc genes were translated using BLASTX and analyzed using InterProScan 5.0^51^ and SignalP 4.1^52^. To supplement these analyses, the Pathogen–Host Interactions (PHI) database (version 4.0, http://www.phi-base.org/)^53^ was used to determine the role of these genes in pathogen virulence.

### Validation using quantitative Reverse Transcription PCR

Quantitative Reverse Transcription Polymerase Chain Reaction (qRT-PCR) was performed to confirm the results of LongSAGE analysis on a subset of genes with primers listed in Tables 4 and 5. Eight chickpea genes were chosen based on the four important biological processes namely ‘Protein metabolism’, ‘Signaling’, ‘Biotic-abiotic stress’ and ‘Hormone metabolism’ indicating differential behavior in SAGE analysis of chickpea DEGs. Root tissues challenged by Foc 2 as one of the race representatives and sampled at eight time-points (0 hpi, 16 hpi, 24 hpi, 2 dpi, 4 dpi, 7 dpi, 14 dpi and 28 dpi) along with their respective controls, were used for this analysis in three biological replicates. For shoot/stem tissues, three time-points *viz*. 24 hpi, 7 dpi and 14 dpi, as representative time-points in early, middle and late stages of Fusarium wilt progression, were used for this analysis in three biological replicates. Primer design, reverse transcription and qRT-PCR were conducted as described in our previous study^8^. Target gene expression was determined using the 2^−ΔΔ^ Ct method and the chickpea *GAPDH* as the reference gene for plant genes and Foc *EF1α* for pathogen genes (Figs S4, S5, S6 and S7).

**Table 4:** Primer sequences of the defense related genes of chickpea and *GAPDH* (used as a reference gene) used for qRT-PCR.

Target gene	Forward primer sequence (5′ to 3′)	Reverse primer sequence (5′ to 3′)	Amplicon size
Mitogen Activated Protein kinase	GGAAGACGTGCGAGAGCTTA	AATCCTGTTGGCTCTGCTCC	93 bp
14-3-3 like protein	TGTGCTGTCTTTGTAAGACTCCT	AAAGGGCATGTCACCTTGCT	89 bp
UDP-glycosyltransferase	GTTGGAAGAGCCGTTTGAGC	TAGCAACATCAACGGGCCAT	98 bp
Auxin binding protein ABP19a	GGCTACCACTGCAAACCTCT	TGCGGCGTTGAATGTGTTTT	96 bp
Linoleate 9S-lipoxygenase	CCCGGTGGTATAATCGGTGG	CCCAAGAAAGAAGTGGCGGT	83 bp
Cystein protease	ATGTGCGGAGGGCTTACAAA	TTTGGGTCTGGTGGTTCAGG	85 bp
DELLA protein	GCAGGAAGCGAATCACAACG	CCAACGAGTCAAACAGCGTC	86 bp
NAC transcription factor	TCCTGTTGGCTTCCAATAACCA	GGTAGAGCTTTGGCTGAGGG	96 bp
Glyceraldehyde phosphate dehydrogenase	CCAAGGTCAAGATCGGAATCA	CAAAGCCACTCTAGCAACCAAA	93 bp

**Table 5:** Primer sequences of the virulence related genes of Foc and *EF1α* (used as a reference gene) used for qRT-PCR.

Target gene	Forward primer sequence (5′ → 3′)	Reverse primer sequence (5′ → 3′)	Amplicon size
Class V Chitin synthase	GGCCTACATCAACTCTGCAAC	GGGCATTATAACGACCGTCTCAA	96 bp
Ubiquitin fusion protein	CAACCCCAATTCGCACCATC	CCGTGAGGGTCTTGACGAAA	96 bp
Chitin synthase 4	CGGATTATGGGGGAAACCATGT	TTGGCCTCAAGAATGTTACCCCTT	99 bp
Worronin body major protein	ACCCGCTCCCCAATTCTATT	GGTTGTACTGAGGGCGAGAT	86 bp
ABC transporter CDR4	GATTCACCCCTTAACCCGCA	CTGTCGAAACCCAGAGCCAT	99 bp
ATP synthase	CAATGTTTGCATGCCCGTCT	CGTTGACACCAGCGAAGATG	98 bp
Β-glucosidase	CTGTTCACCGAGTGCATCCT	AAATCACCGTTGCCATTGCC	91 bp
60S ribosomal protein	GTGCCCTCAAGTACGTCGAA	ATTGACGGAGTTCCCAGCAG	93 bp
*EF1α*	AGCTCGGTAAGGGTTCCTTC	TCCAGAGAGCAATATCGATGG	93 bp

### Data Availability

The LongSAGE sequencing data have been deposited in Gene Expression Omnibus in NCBI under the accession numbers: GSM2301186, GSM2301187, GSM2301188 and GSM2301189.

## Electronic supplementary material

Tables S1-S11

Supplementary Figures
