# Supplementary material for: Chickpea-Fusarium oxysporum interaction transcriptome reveals differential modulation of plant defense strategies
Source: Sci Rep. 2017 Aug 10;7:7746. doi: 10.1038/s41598-017-07114-x (PMC5552786; doi:10.1038/s41598-017-07114-x)
Supplement: Supplementary file 2 — Supplementary Figures [file 41598_2017_7114_MOESM2_ESM.pdf]

Supplementary Figures

Manuscript title: Chickpea-*Fusarium oxysporum* interaction transcriptome reveals differential modulation of plant defense strategies

Authors: Medha L. Upasani, Bhakti M Limaye, Gayatri S. Gurjar, Sunitha Manjari K., Rajendra R. Joshi, Narendra Y. Kadoo, Vidya S. Gupta

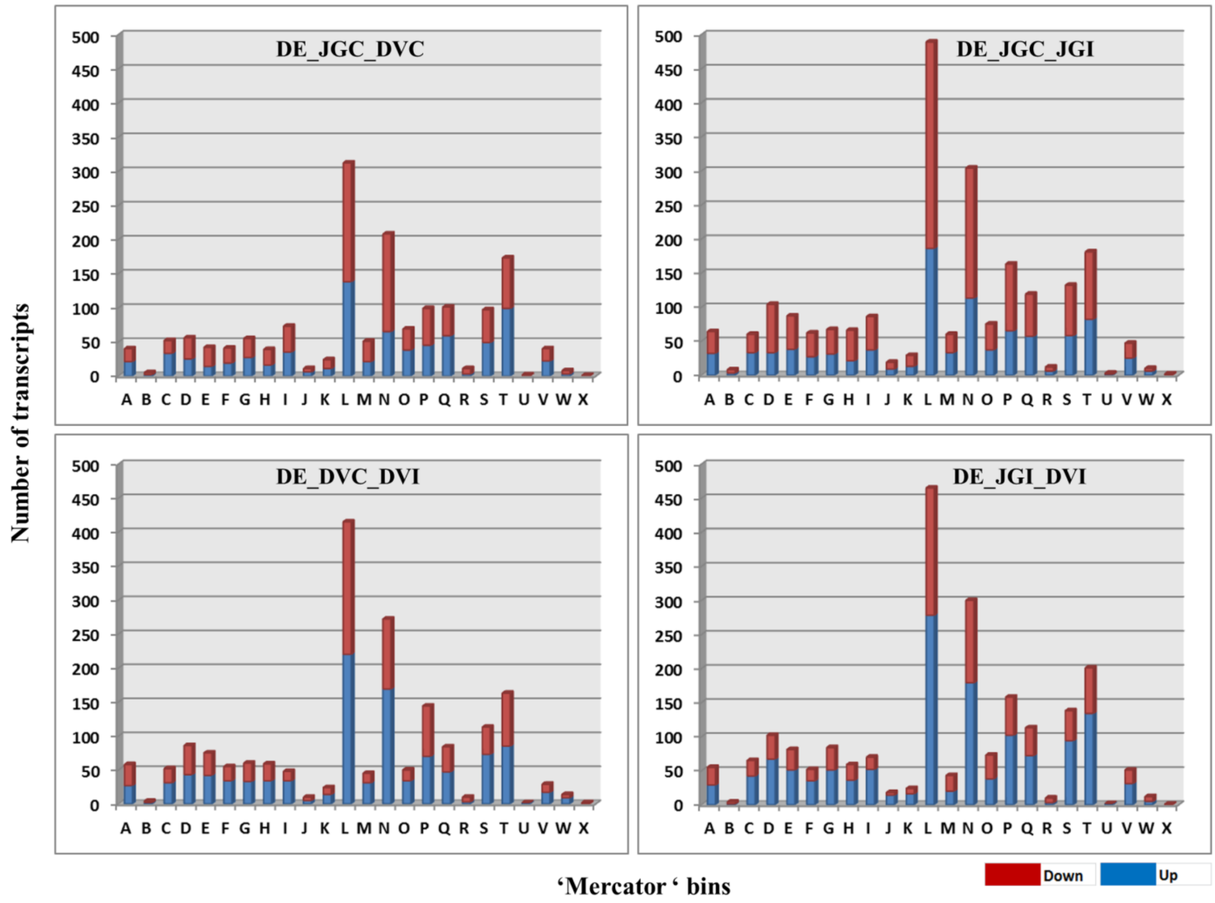

Fig. S1: The distribution of functional classes among up- and down-regulated chickpea genes in four Differential Gene Expression (DGE) datasets. The annotation tool 'Mercator' based assignment of the genes to different functional classes (bins) is represented on X axis (A - Amino acid metabolism; B - Biodegradation of Xenobiotics; C - Cell wall related; D - Cell E – Development; F - DNA metabolism; G - Hormone metabolism; H - Lipid metabolism; I - CHO metabolism; J - ATP synthesis; K - Nucleotide metabolism; L - Protein metabolism; M – Photosynthesis; N - RNA metabolism; O - Secondary metabolism; P – signaling; Q – Stress; R - Tetrapyrrole synthesis; S – Transport; T – Miscellaneous; U - Polyamine metabolism; V – Redox; W - Vitamin metabolism; X - S-assimilation). Y axis denotes number of transcripts belonging to a particular bin.

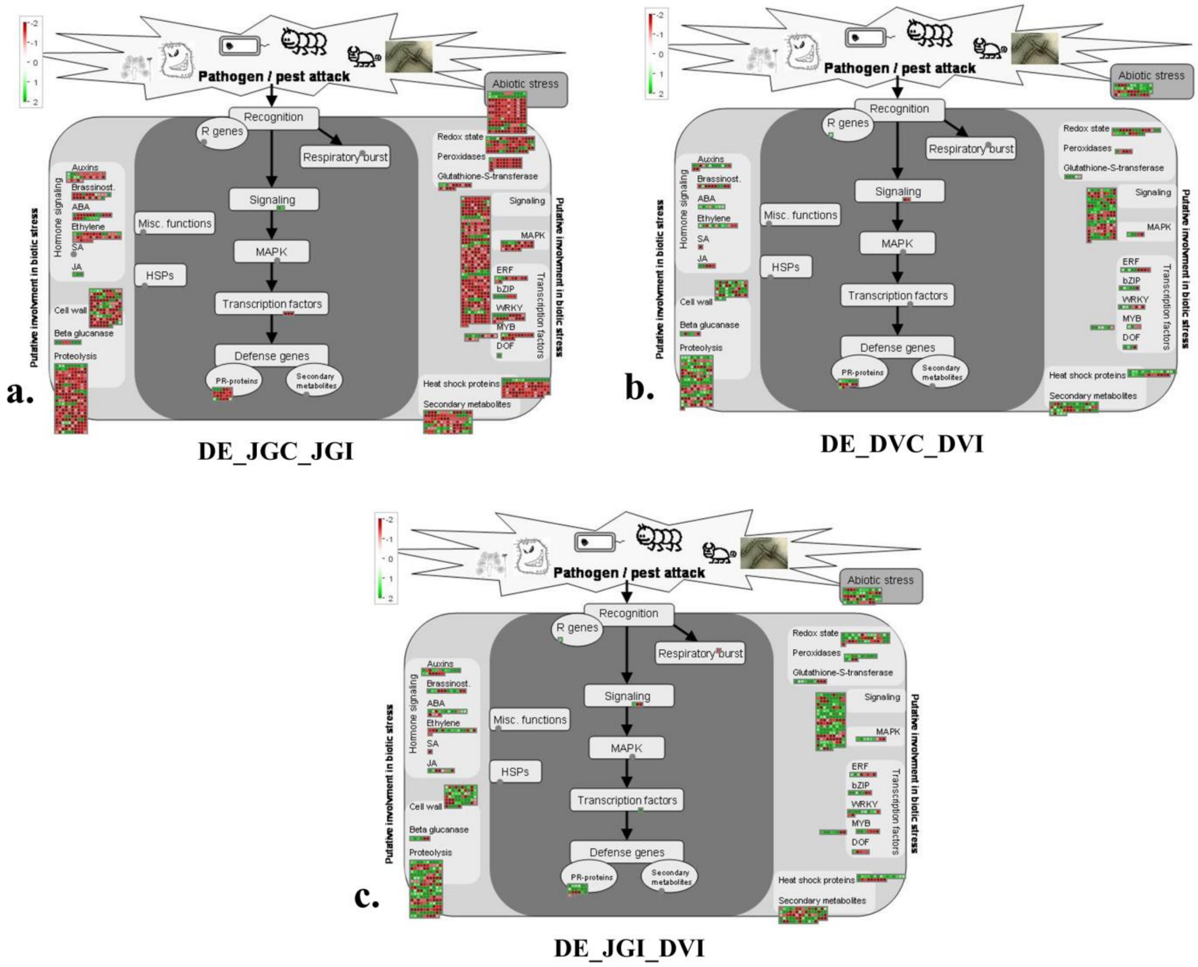

Fig. S2: MapMan presentation of DEGs (chickpea) of the datasets DE_JGC_JGI, DE_DVC_DVI and DE_JGI_DVI under 'Stress' category. MapMan annotation software was used to display DEGs of the three datasets represented during stress namely (A) DE_JGC_JGI, (B) DE_DVC_DVI and (C) DE_JGI_DVI. The color change from dark red (+2 LFC) to dark green (-2 LFC) through white (0 LFC) indicates the differential expression ranging from 4 fold up-regulation to 4 fold down-regulation.

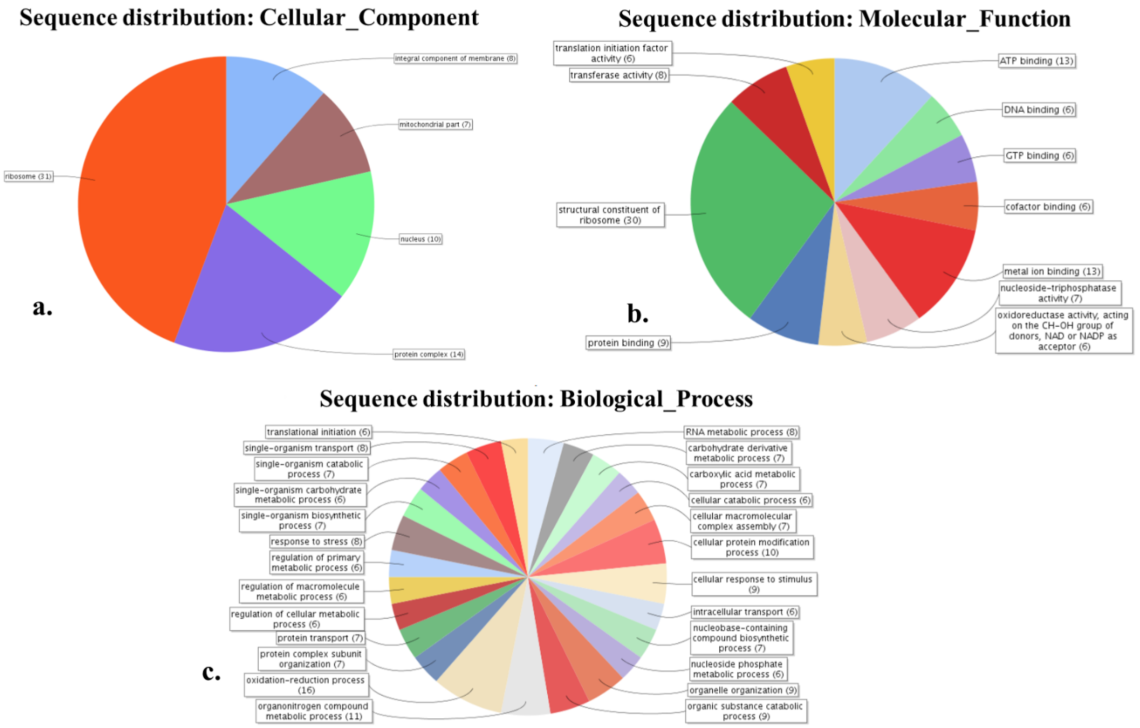

Fig. S3: Gene enrichment analysis of *Fusarium* genes expressed exclusively in JG I. Distribution of 533 *Fusarium* genes expressed only in JG I as A. Cellular Component (CC) B. Molecular Function (MF) and C. Biological Process (BP) based on Blast2GO analysis.

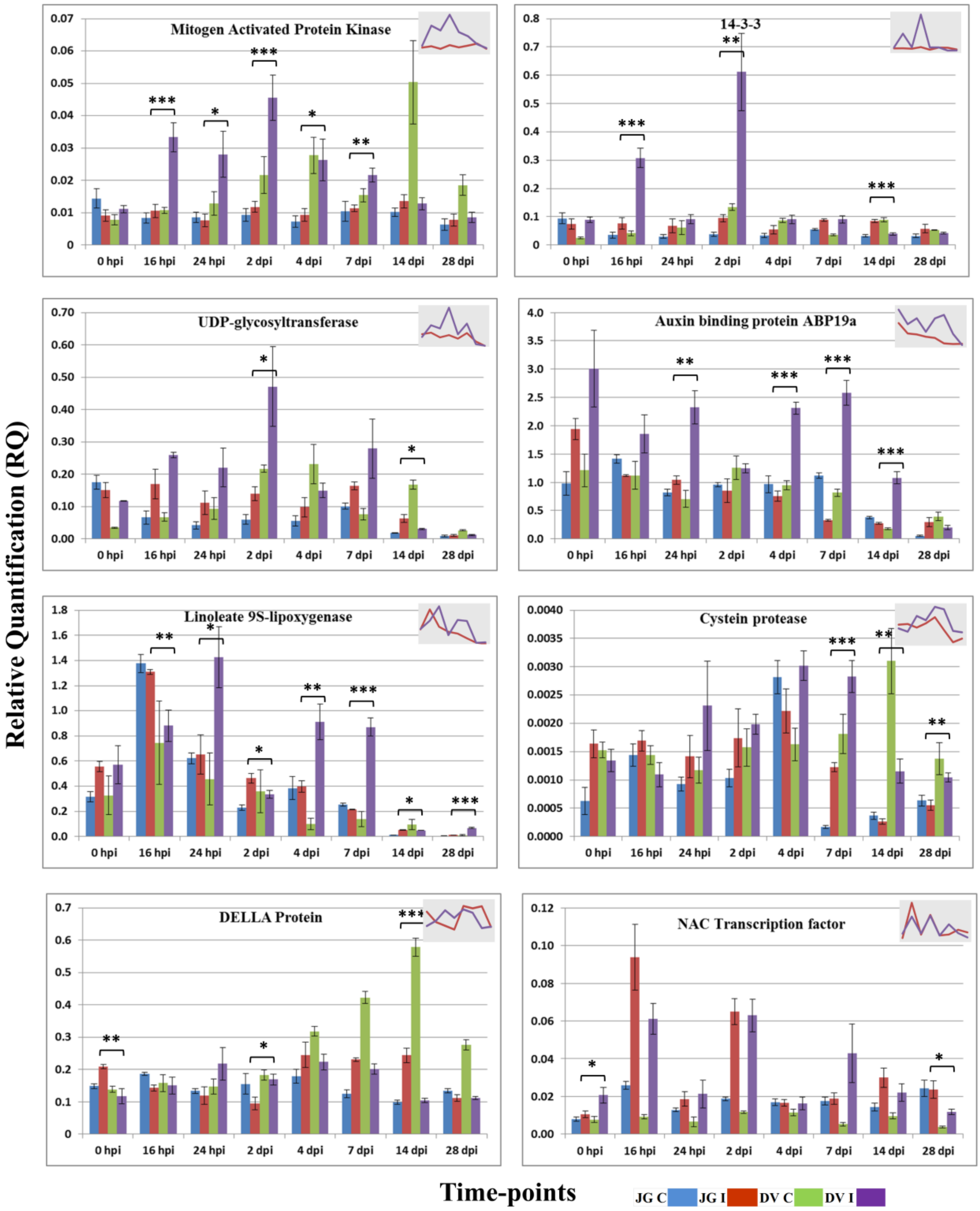

Fig. S4: Expression patterns of eight chickpea defense related genes in root tissue at eight different time-points using quantitative reverse transcriptase PCR. The inset in each graph depicts expression of the gene in JGI and DVI across the time-points.

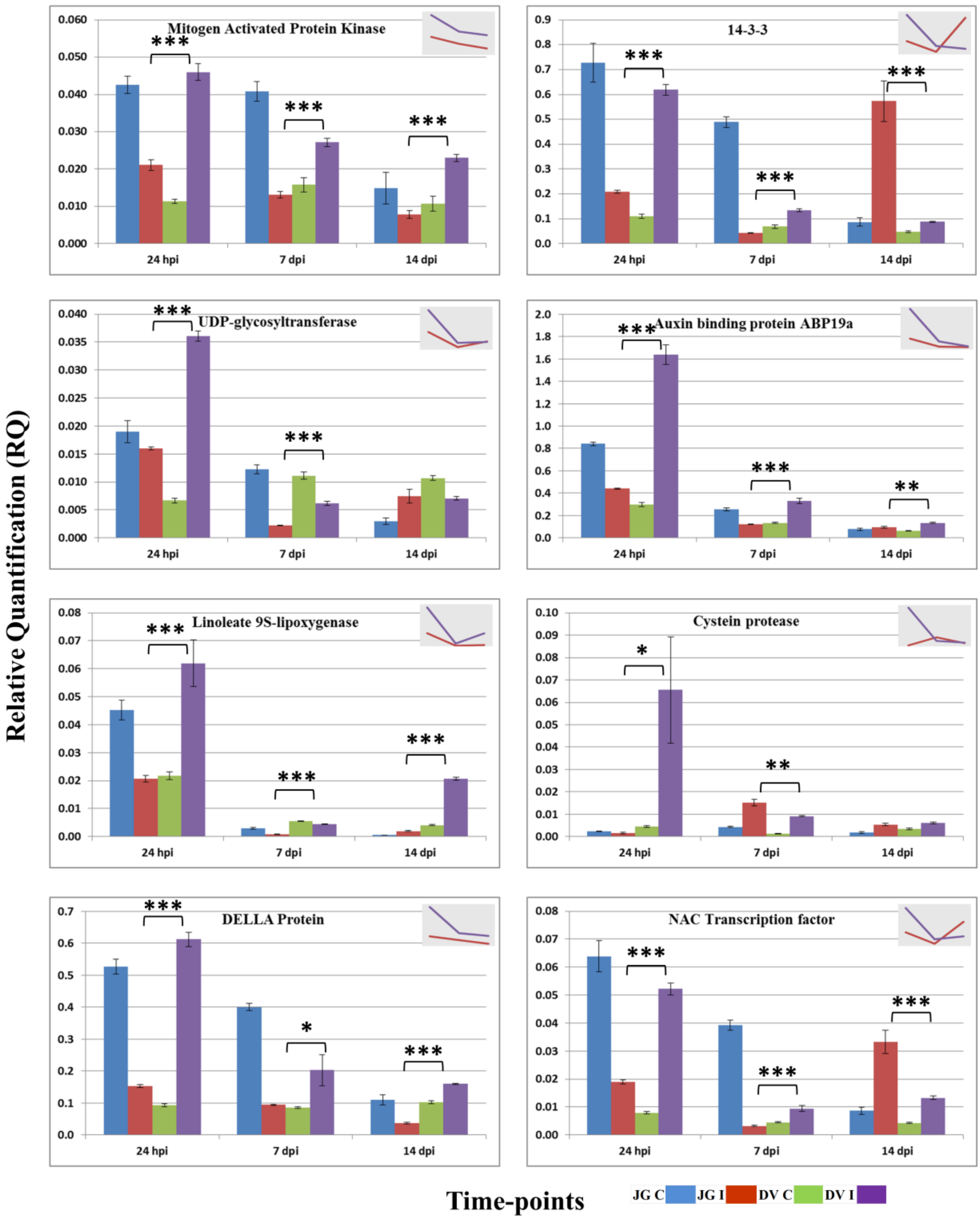

Fig. S5: Expression patterns of eight chickpea defense related genes in shoot tissue at three different time-points using quantitative reverse transcriptase PCR. The inset in each graph depicts expression of the gene in JGI and DVI across the time-points.

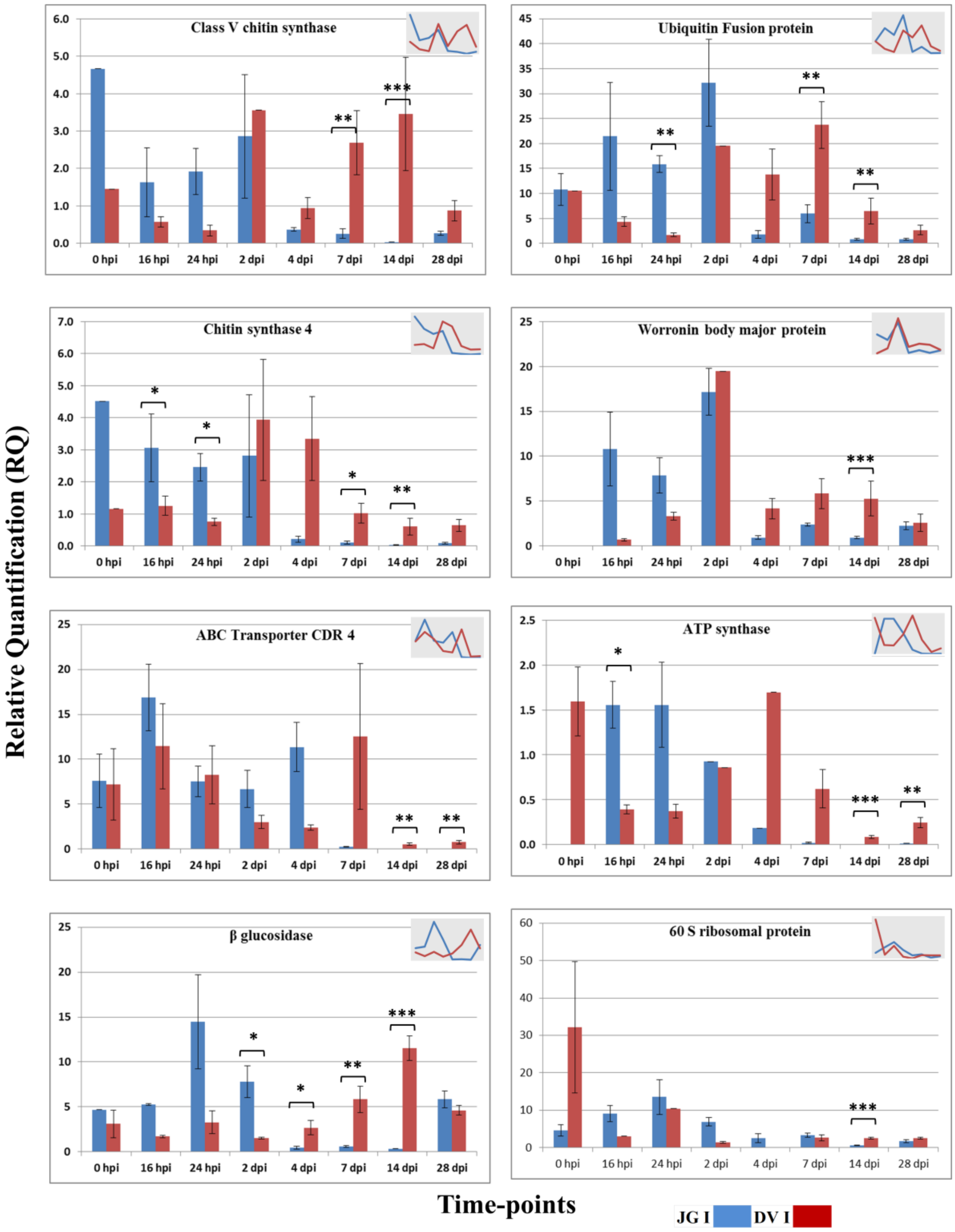

Fig. S6: Expression patterns of eight pathogen virulence related genes in root tissue at eight different time-points using quantitative reverse transcriptase PCR. The inset in each graph depicts expression of the gene in JGI and DVI across the time-points.

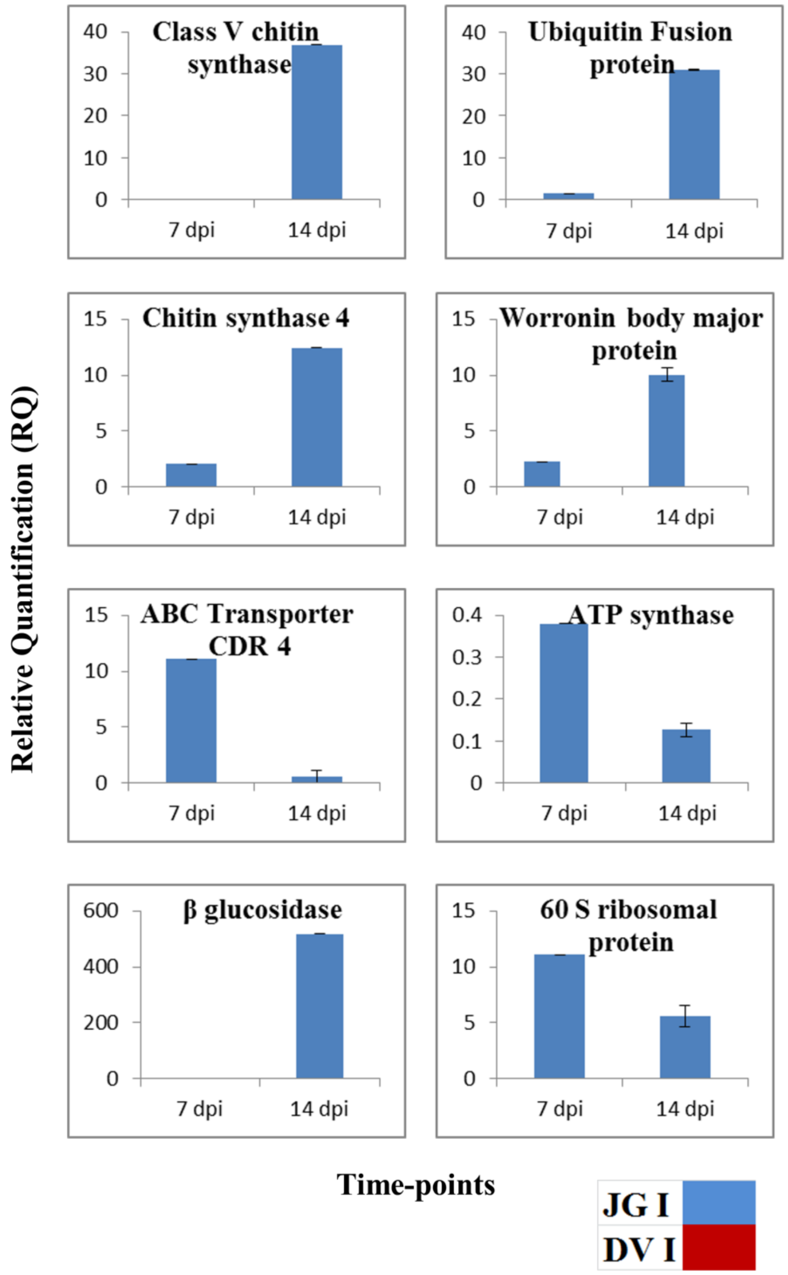

Fig. S7: Expression patterns of eight pathogen virulence related genes in shoot tissue at three different time-points using quantitative reverse transcriptase PCR. The inset in each graph depicts expression of the gene in JGI and DVI across the time-points.

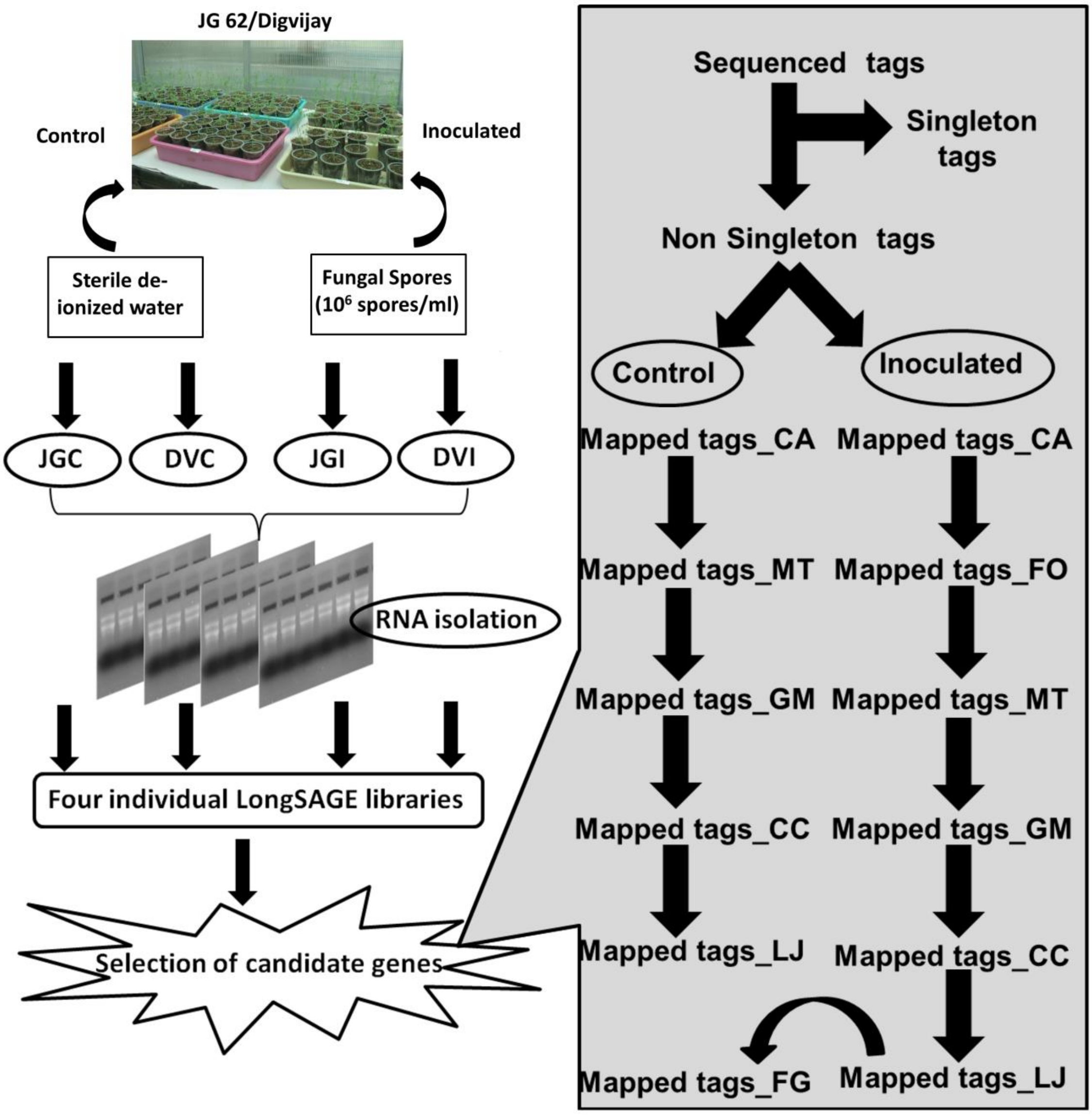

Fig. S8: Experimental design of the present study. Susceptible (JG 62) and resistant (Digvijay) cultivars of chickpea were raised in greenhouse. Seedlings treated with sterile deionized water (un-inoculated plants) served as control while those inoculated with Foc 1, 2 and 4 served as treated. Total four SAGE libraries were constructed for each treatment: susceptible control (JG C), susceptible inoculated (JG I), resistant control (DV C) and resistant inoculated (DV I). Analysis of all four libraries with the respective available transcriptomes led to the identification of candidate chickpea as well as *Fusarium* genes.

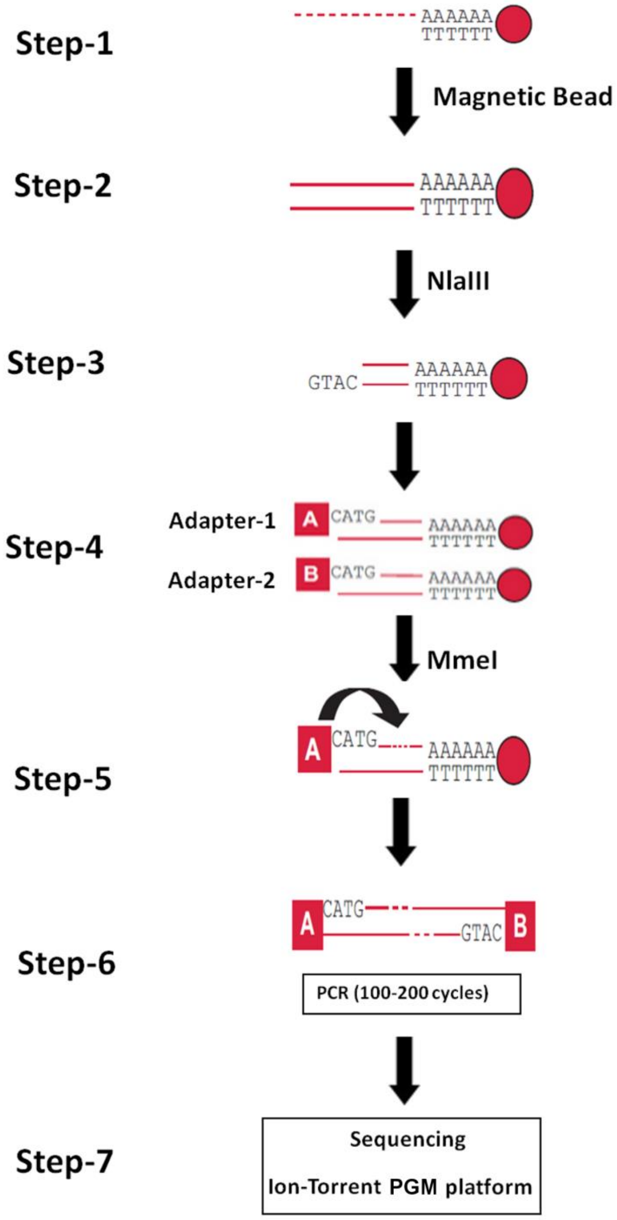

Fig. S9: Schematic representation of LongSAGE library construction protocol. The Figure shows seven consecutive steps for the construction of LongSAGE library starting with mRNA. mRNA captured on magnetic beads is reverse transcribed into cDNA. An anchoring enzyme NlaIII cleaves cDNA generating 4 bp overhang 'CATG'. Adapters A and B are ligated to this cleaved cDNA pool and another restriction enzyme MmeI is used. It cleaves cDNA generating an adapter-tag pool in supernatant which are then ligated to form ditags. Ditags are PCR amplified and sequenced using an Ion-Torrent platform.
